# Mechanisms of angiogenesis in tumour

**DOI:** 10.3389/fonc.2024.1359069

**Published:** 2024-03-25

**Authors:** Run Zhang, Yutong Yao, Hanwei Gao, Xin Hu

**Affiliations:** China–Japan Union Hospital of Jilin University, Jilin University, Changchun, China

**Keywords:** sprouting angiogenesis, anti-angiogenesis therapy, vasculogenic mimicry, vascular co-option, tumour microenvironment

## Abstract

Angiogenesis is essential for tumour growth and metastasis. Antiangiogenic factor-targeting drugs have been approved as first line agents in a variety of oncology treatments. Clinical drugs frequently target the VEGF signalling pathway during sprouting angiogenesis. Accumulating evidence suggests that tumours can evade antiangiogenic therapy through other angiogenesis mechanisms in addition to the vascular sprouting mechanism involving endothelial cells. These mechanisms include (1) sprouting angiogenesis, (2) vasculogenic mimicry, (3) vessel intussusception, (4) vascular co-option, (5) cancer stem cell-derived angiogenesis, and (6) bone marrow-derived angiogenesis. Other non-sprouting angiogenic mechanisms are not entirely dependent on the VEGF signalling pathway. In clinical practice, the conversion of vascular mechanisms is closely related to the enhancement of tumour drug resistance, which often leads to clinical treatment failure. This article summarizes recent studies on six processes of tumour angiogenesis and provides suggestions for developing more effective techniques to improve the efficacy of antiangiogenic treatment.

## 1 Introduction

Tumours satisfy their need for oxygen and nutrients by establishing new blood vessels to perform other metabolic functions (1). Tumour angiogenesis was initially defined as vascular endothelial cells proliferating and migrating based on existing capillaries or veins so that the original blood vessels form a new vascular system (2). Following decades of research, however, it has been shown that tumour angiogenesis is a complicated dynamic process involving various cell types and complex signalling networks. The tumour microenvironment (TME) encompasses the internal and external surroundings of tumour cells during their growth and metastasis. It consists of various components, including tumour cells, endothelial cells (ECs), other tissue-resident cells, including adipocytes, tumour-associated macrophages (TAMs), stromal cells such as infiltrating leukocytes, cancer-associated fibroblasts (CAFs), along with bone marrow-derived cells (BMDCs) (3). It plays a crucial role in inducing immune suppression, immune tolerance, and tumour angiogenesis. Further studies have revealed that high levels of angiogenesis depend on the expression of vascular growth factors and the regulation of various angiogenesis-related signalling pathways, highlighting the significance of angiogenesis-related signalling pathways in antitumour therapeutic strategies.

**Figure 1:**
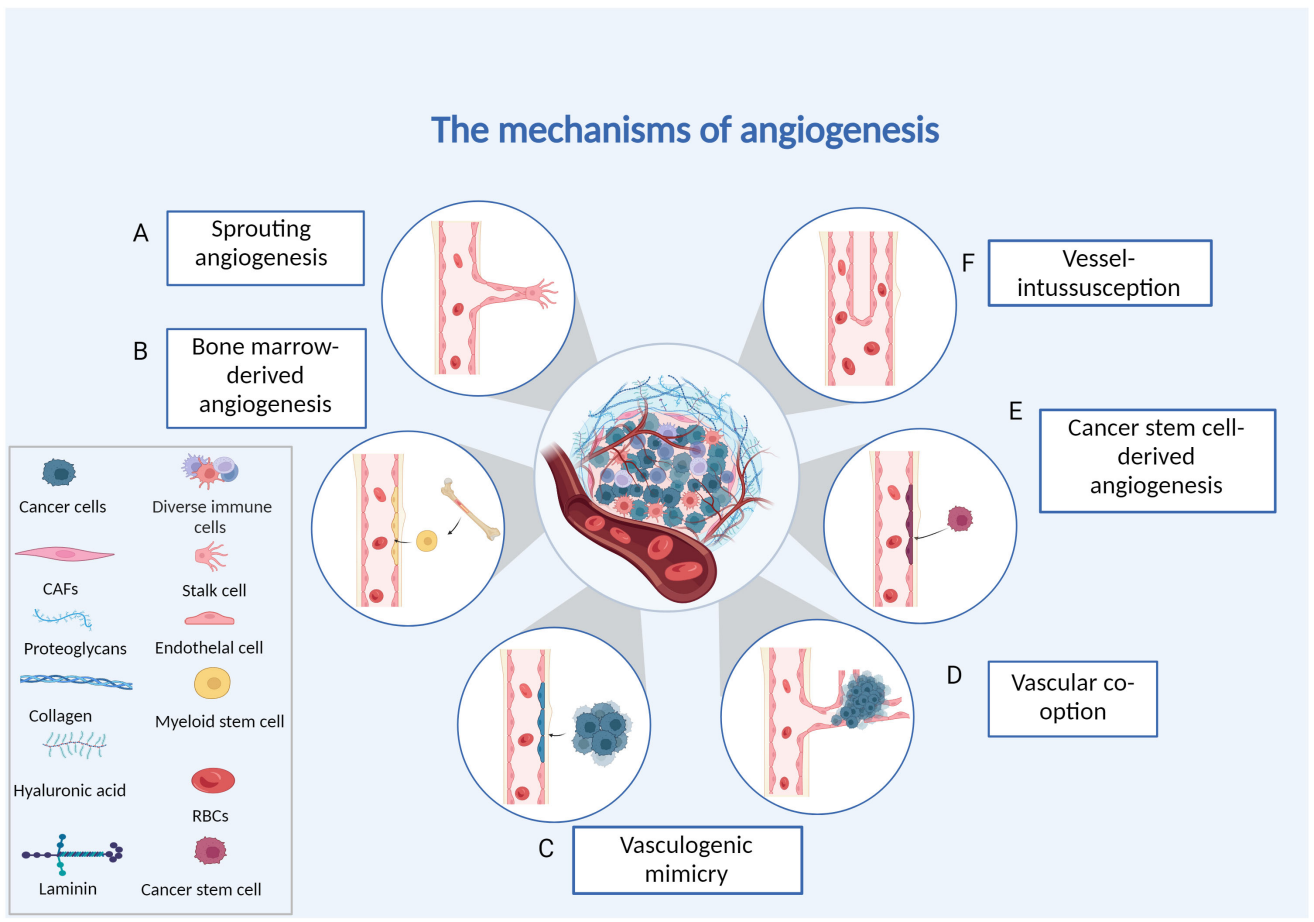
Different forms of tumour angiogenesis. **(A)** Sprouting angiogenesis: endothelial cells proliferating and migrating based on existing capillaries. **(B)** Bone marrow-derived angio-genesis: bone marrow-derived endothelial progenitor cells differentiate into endothelial cells to form blood vessels. **(C)** Vasculogenic mimicry: the vascular channels are made up of tumour cells. **(D)** Vessel co-option: invading normal tissues, tumour cells make use of the vascular system already in place. **(E)** Cancer stems cell-derived angiogenesis: tumour cells with stemness differentiate into endothelial cells. **(F)** Intussusceptive angiogenesis: endothelial cells split into two vessels without proliferating. Created with BioRender.com.

With in-depth exploration of the angiogenesis mechanism, many anti-angiogenesis drugs (AADs) have been discovered and applied in clinical practice and have achieved apparent effects in the treatment of various tumours (4). However, despite the advancements in antiangiogenic therapy that have significantly improved the lives of many cancer patients, resistance to antiangiogenic medication often emerges during clinical treatment, leading to suboptimal therapeutic outcomes and treatment failure. Increasing evidence shows that complex angiogenic mechanisms enable tumours to evade antiangiogenic therapy (5–8). Currently, research on angiogenesis has focused on the mechanism of sprouting angiogenesis, while other mechanisms of angiogenesis remain poorly understood.In this review, we summarized the findings related to six primary cellular mechanisms of tumour angiogenesis (**Figure 1**): (1) sprouting angiogenesis (SA), (2) vasculogenic mimicry (VM), (3) vessel intussusception, (4) vascular co-option, (5) cancer stem cell-derived angiogenesis and (6) bone marrow-derived angiogenesis. Additionally, we also discuss the development and current challenges associated with anti-angiogenic therapy. It is our hope that further studies will enhance researchers’ understanding of angiogenesis mechanisms and regulation, facilitating the provision of more effective and individualized antiangiogenic therapies.

## 2 Different mechanisms of angiogenesis

### 2.1 Sprouting angiogenesis

SA, which involves the development of new capillaries from pre-existing blood vessels, has long been considered the predominant process underlying tumour vascularization (9). This intricate process is subject to multiple regulatory mechanisms governing vascular remodelling and maturation. These mechanisms encompass crucial signalling pathways, including: (1) The fibroblast growth factor (FGF) and its receptor (FGFR)signalling pathway: FGF and FGFR play pivotal roles in orchestrating angiogenesis by stimulating the growth and development of blood vessels. (2) The delta-like ligand 4 (DLL4)/Notch signalling pathway: DLL4 interacts with the Notch receptor, influencing cell fate decisions during angiogenesis and promoting vessel sprouting and branching. (3) The vascular endothelial growth factor (VEGF) and its receptor (VEGFR) signalling pathway: VEGF and VEGFR constitute a fundamental axis in angiogenesis, facilitating the formation of new blood vessels and promoting tumour angiogenesis. (4) The platelet-derived growth factor (PDGF) and its receptor (PDGFR) signalling pathway: PDGF contributes to the growth and maturation of blood vessels, playing a role in the tumour’s vascularization process (10–12). These signalling pathways are interconnected and interact to enhance the proliferation of vascular epidermal cells and induce tumour angiogenesis, creating favourable circumstances for malignant tumour development, invasion, and metastasis.

ECs demonstrate the ability to differentiate into two distinct phenotypes within the context of angiogenesis: tip cells, characterized by their capacity to migrate in response to a concentration gradient of the signalling molecule VEGF, and stalk cells, which are proficient in proliferative activities (13). In the intricate process of angiogenesis, the tip cells play a pivotal role by navigating toward the source of VEGF, guiding the extension of new vascular branches. These branches subsequently fuse with other neighbouring branches, culminating in the formation of a continuous vascular channel. This nascent vascular channel serves as the foundation for the subsequent development of a sophisticated network of blood vessels, encompassing capillaries, arterioles, and venous vessels. This orchestrated process significantly amplifies the extent of tumour angiogenesis (14), contributing to the increased supply of oxygen and nutrients to the tumour microenvironment, ultimately facilitating the progression and sustenance of malignant tumours.

Upon reaching a certain stage of growth, solid tumours are exposed to a hypoxic environment, which is considered a hallmark feature of tumour growth. Solid tumours generate a significant amount of Hypoxia-inducible factor 1 (HIF-1) under hypoxic conditions, and HIF-1 plays a crucial role in tumour progression, metastasis, and adverse prognosis. Research indicates that HIF-1 can activate the transcription of hypoxia-inducible genes by targeting genes’ promoters with a common DNA sequence known as hypoxia response elements (HREs). When induced, these HREs are activated, leading to the excessive secretion of various factors that promote angiogenesis (15). Essentially, the upregulation of HIF-1 in the hypoxia response plays a critical role in orchestrating molecular events that promote new blood vessel formation, providing essential oxygen and nutrients for the survival and development of tumours. Under hypoxic conditions, HIF-1 upregulates growth factors such as VEGF and FGF, promoting endothelial cell vascular permeability, endothelial cell growth, and proliferation. Furthermore, it can activate other growth factors, including PDGF, TGF, and the transcription of angiopoietins (Ang), facilitating the release of angiogenic factors by cancer cells.

**Figure 2:**
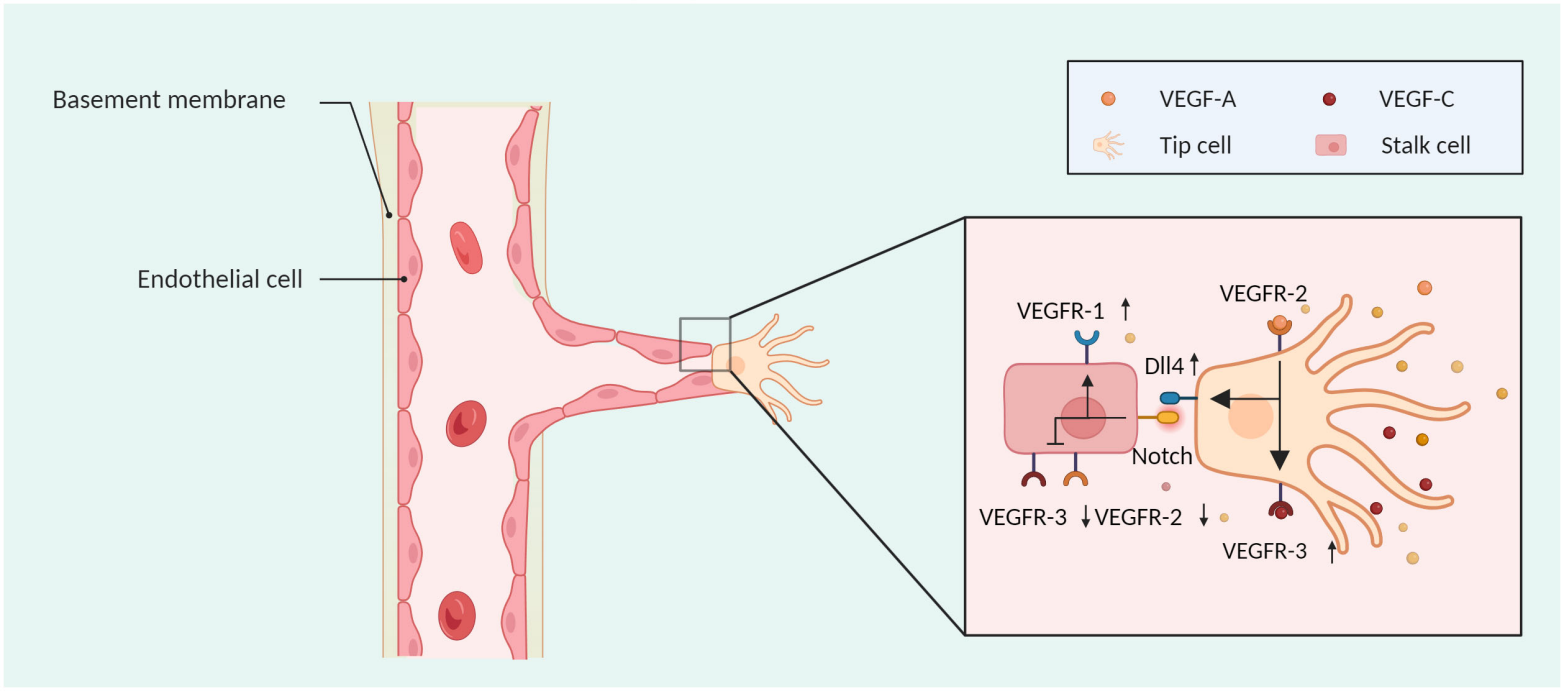
The sprouting mode of angiogenesis. Tip cells can move in response to a VEGF concentration gradient, and stalk cells can proliferate. VEGF-A activates VEGFR-2, which leads to the upregulation of VEGFR-3 and Dll4 in tip cells and activation of the NOTCH pathway in neighbouring stalk cells, wherein VEGFR-2 and VEGFR-3 are downregulated, and VEGFR-1 is upregulated. Created with BioRender.com.

VEGF is a proangiogenic factor that specifically targets vascular endothelial cells and is involved in a variety of events, such as tumour development, invasion, and angiogenesis. Members of the VEGF family include VEGF-A, VEGF-B, VEGF-C, VEGF-D, and placental growth factor (PIGF) (16). VEGF-A activates VEGFR-2, which leads to the upregulation of VEGFR-3 and Dll4 in tip cells and activation of the Notch pathway in neighbouring stalk cells. This process results in the downregulation of VEGFR-2 and VEGFR-3 and upregulation of VEGFR-1, maintaining the balance between tip and stalk cells (**Figure 2**). The downstream pathways of the p38-mitogen-activated protein kinase (p38/MAPK) and AKT-phosphatidylinositol-3 kinases (PI3K/AKT) are activated upon VEGF-A binding to VEGFR-2 on the surface of ECs (17), causing ECs to proliferate and migrate, producing a large number of proangiogenic proteases that increase the permeability of the vessel barrier.

Once ECs have sprouted and aggregated to form a vascular prototype, they release factors to attract pericytes. Notably, heparin-binding EGF-like growth factor stimulates pericyte proliferation, while transforming growth factor beta 1/2 (TGFβ1/2) initiates pericyte recruitment (18). Additionally, by attracting pericytes and tumour-associated fibroblasts, PDGF is essential in the process of tumour angiogenesis. The PDGF family includes PDGF-A, PDGF-B, PDGF-C, and PDGF-D (19). PDGF binds PDGFR and promotes receptor dimerization, thereby initiating signalling, including the Ras-mitogen-activated protein kinase (RAS/MAPK) and PI3K pathways (20). PDGF-AB stabilizes the newly formed tumour vascular system by recruiting pericytes (21). PDGF-BB regulates VEGFR2 signalling and endothelial proliferation to prevent vascular abnormalities due to high levels of VEGF (22).

Ang induces tumour-endothelial cell interactions to regulate tumour angiogenesis. Ang-1, Ang-2, Ang-3, and Ang-4 are members of the Ang family, while Tie1 and Tie2 are their corresponding tyrosine kinase receptors (23). Ang-1-mediated signalling mechanisms involving Tie2 phosphorylation have the potential to maintain tight endothelial cell interactions (24). A reduction in vascular sprouting and the induction of tumour neovascularization can be achieved by blocking Tie/Ang-2 (11). In addition, the Ang-2/Tie signalling and VEGF signalling pathways synergistically promote tumour neo-angiogenesis, blockade of Ang-2 and VEGF delays tumour growth and enhances survival benefits through reprogramming of tumour-associated macrophages toward an antitumour phenotype as well as by pruning immature tumour vessels (25). Pericytes release Ang-1, which binds to endothelial cell TIE2 receptors, thereby efficiently inhibiting endothelial cell proliferation and promoting angiogenesis stability through downstream signalling pathways, such as Calpain, protein kinase B, and forkhead box O3A (26).

**Figure 3:**
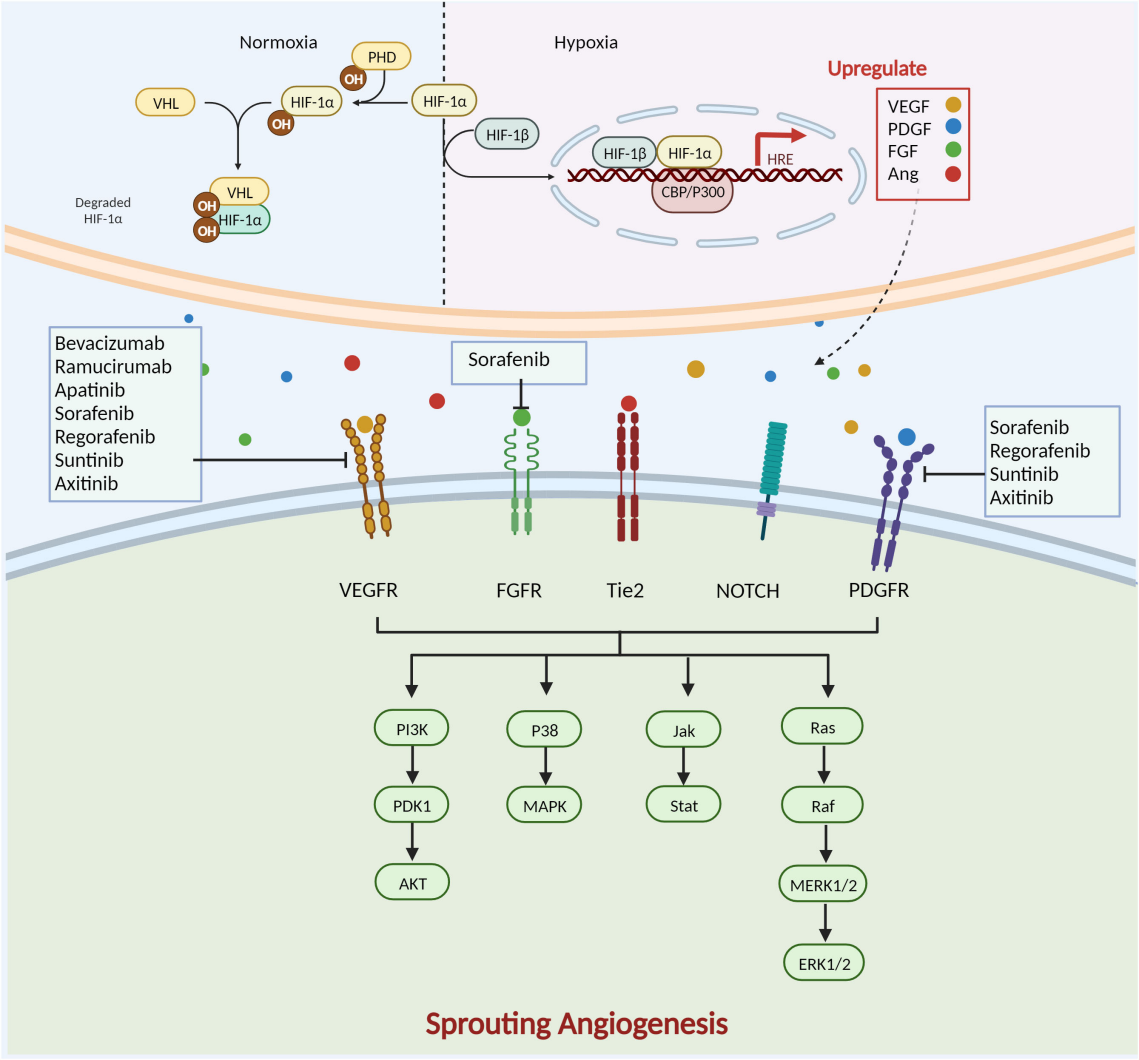
Complex mechanisms of SA formation and anti-angiogenesis therapy. HIF-1 is composed of two subunits: HIF-1α and HIF-1β. In normoxia, HIF-1α is hydroxylated by prolyl hydroxylase (PHD) in the cyto-plasm in an oxygen-dependent manner. It then forms a complex with the von Hippel-Lindau protein (VHL) and other proteins, is ubiquitinated and degraded by the proteasome. During hypoxia, lack of oxygen-dependent hydroxylation and proteasomal degradation of HIF-1α leads to its accumulation. It then forms a dimer with HIF-1β and enters the nucleus, interacts with cAMP response element-binding protein and p300 protein (CBP and p300), and binds to HRE to activate the transcription of hundreds of genes, including VEGF, PDGF, Ang, and FGF.VEGF/VEGFR activates the downstream p38/MAPK and PI3K/AKT path-ways. Ramucirumab, sorafenib, regorafenib, sunitinib, axitinib, apatinib and bevacizumab de-crease angiogenesis by targeting VEGF/VEGFR. PDG/PDGFR causes dimerization of PDGFR-α and PDGFR-β of different subtypes of the receptor and activation of specific downstream path-ways. Sorafenib, regorafenib, sunitinib, and axitinib also target PDGFR to inhibit angiogenesis. The Notch pathway mediates angiogenesis through activation of Ras. FGF/FGFR activates downstream PI3K/AKT, Janus kinase/signal transducer and activator of transcription (Jak/Stat), and other signalling pathways to regulate tumour angiogenesis. Sorafenib inhibits angiogenesis by targeting FGFR. Created with BioRender.com.

Anti-angiogenic targeted therapies inhibit the proliferation, invasion, and metastasis of malignant tumours by disrupting proangiogenic signalling between tumour cells and ECs (**Figure 3**). Currently, the clinical application of AADs mainly includes antiangiogenic macromolecular monoclonal antibodies, small-molecule antiangiogenic tyrosine kinase inhibitors, and antiangiogenic-related molecule inhibitors. The most prevalent macromolecular monoclonal medicines are bevacizumab and ramucirumab, and the primary targets of these medications are VEGF-A and VEGFR. Bevacizumab as a first-line regimen for metastatic colorectal cancer (mCRC) has been shown in clinical trials to extend the median overall survival and progression-free survival of mCRC patients (27). However, due to its single targeting that is typically combined with chemotherapeutic drugs, bevacizumab, when combined with platinum-based therapies, has a considerable PFS advantage in metastatic non-small cell lung cancer (NSCLC) (28).Tyrosine kinase inhibitors, such as sorafenib, sunitinib, and pazopanib, control angiogenesis by preventing the function of receptor tyrosine kinases that accelerate angiogenesis. Since the approval of Sorafenib in 2007, significant progress has been made in the treatment of hepatocellular carcinoma (HCC) (29). Studies of combination therapy with molecule-targeted drugs and immune checkpoint inhibitors are underway, promising to benefit more patients (30). Currently, a Phase III trial has been conducted to effectively treat neovascular age-related macular degeneration (nAMD) by using faricimab, an antibody targeting Ang/Tie and VEGFR pathway (31).

### 2.2 Vascular co-option

The process by which tumour cells invade normal tissues and utilize the existing circulatory system to gather nutrition and oxygen is known as vascular co-option (32). Pezzella first discovered in 1997 that tumours could also grow in nonangiogenic ways in non-small cell lung cancer cells (33). Further studies have revealed that vascular co-option occurs in lung cancer, renal cell carcinoma (RCC), colon cancer, glioma and breast cancer (34–40). Under a light microscope, the characteristic pathological morphology of vascular co-option may be observed by staining tissue sections with the commonly used haematoxylin and eosin method (41). By quantitatively analysing CT and MRI images, angiogenic tumours can be distinguished from tumours that utilize vascular gain to accurately distinguish between these two phenotypes (41). Vascular co-option is characterized by intact vascular architecture of normal tissue within the tumour and tumour uptake of normal vessels surrounding the tissue rather than neovascularization, inflammation, or fibrosis (42). In contrast, tumour angiogenesis usually manifests as disorganization.

Angiogenesis is highly dynamic in time and space, with some tumour not only budding through vascular co-option but also utilizing sprouting angiogenesis to derive nutrients from them. There are two main ways for blood vessel absorption between tumour and adjacent tissues: (1) malignant cells replace normal epithelial cells (2) tumour cells invade the perivascular matrix (43). The molecules involved in the various stages of vascular co-option formation have been summarized by Ying Shao (44). The occurrence of vascular co-option seems to be related to the type of tumour and metastatic site. By analysing 164 cases of lung metastases (34), it was observed that 91.3% of breast cancer, 98.2% of colorectal cancer, and 62.3% of kidney cancer metastases exhibited some degree of vascular absorption growth. Additionally, 71.7% of breast cancer, 78.9% of colorectal cancer, and 37.7% of kidney cancer metastases had the vascular uptake growth pattern as their predominant pattern. Furthermore, evaluation of haematoxylin and eosin staining tissue sections from 45 cases of liver metastases found that a vascular resorption pattern was present in 96% of breast cancer liver metastases compared with only 32% of colon cancer metastases (45). Compared to renal and colorectal malignancies, vascular co-option growth patterns are more prevalent in breast cancer, and most colorectal cancer liver metastases induce a wound-healing response through angiogenesis. These data suggest that vascular co-option often occurs in breast cancer metastases to the lung and liver, which may help to explain why VEGFR antiangiogenic medication has had very little efficacy in treating metastatic breast cancer. The ability of tumour cells to resist such therapies through a mechanism known as vascular co-option is becoming increasingly obvious. Research by Victoria has demonstrated that although the drug sunitinib can inhibit the growth of subcutaneous xenografts, tumours that metastasize to the lung often utilize vascular co-option (34). Further evidence indicates that patients with HCC treated with sorafenib may experience a shift from vascular sprouting to vascular uptake driven by tumour cells (5). Additionally, intracranial glioblastoma tumours exhibit slower growth in response to anti-VEGF treatment but appear to adapt to angiogenesis inhibition by co-opting the host vasculature (6). The co-option of pre-existing brain vasculature contributes to malignant development without the production of sprouting angiogenesis, as further demonstrated by a model of malignant melanoma brain metastases (46).

Due to the lack of accurate marker analysis of vascular co-option, which can only be performed by analysing tissue slices of patients, studies on the mechanisms of vascular co-option are unclear. Current studies have shown that vascular co-option is related to the epithelial mesenchymal transformation (EMT) process and vascular adhesion of tumours. EMT refers to a phenotype in which ECs lose their polarity through a specific process to obtain a higher ability to invade and migrate (47). Tumour cells are more likely to break through the basement membrane of blood vessels and enter the blood vessels to absorb nutrients and oxygen. Therefore, the occurrence of vascular co-option in tumours is also correlated with the factors and signalling channels related to EMT process. As a known signalling pathway in EMT process, the Wnt signalling pathway is involved in tumour EMT process and plays a crucial role in the occurrence and development of tumours (48). In glioma, treating cells with LGK974, an inhibitor of the Wnt signalling pathway, significantly reduced Wnt7a expression *in vivo*, preventing tumour cell migration along vascular endothelial cells and contact with blood vessels (36). Serine proteinase 2 (35), runt-related transcription factor-1/actin-related protein 2/3 complex (49), fibroblast activation protein α (50) are signalling molecules involved in EMT. The deletion of these genes will lead to changes in cell motility and promote tumour selection of vascular co-option mode mediating the malignant development of cancer.

**Figure 4:**
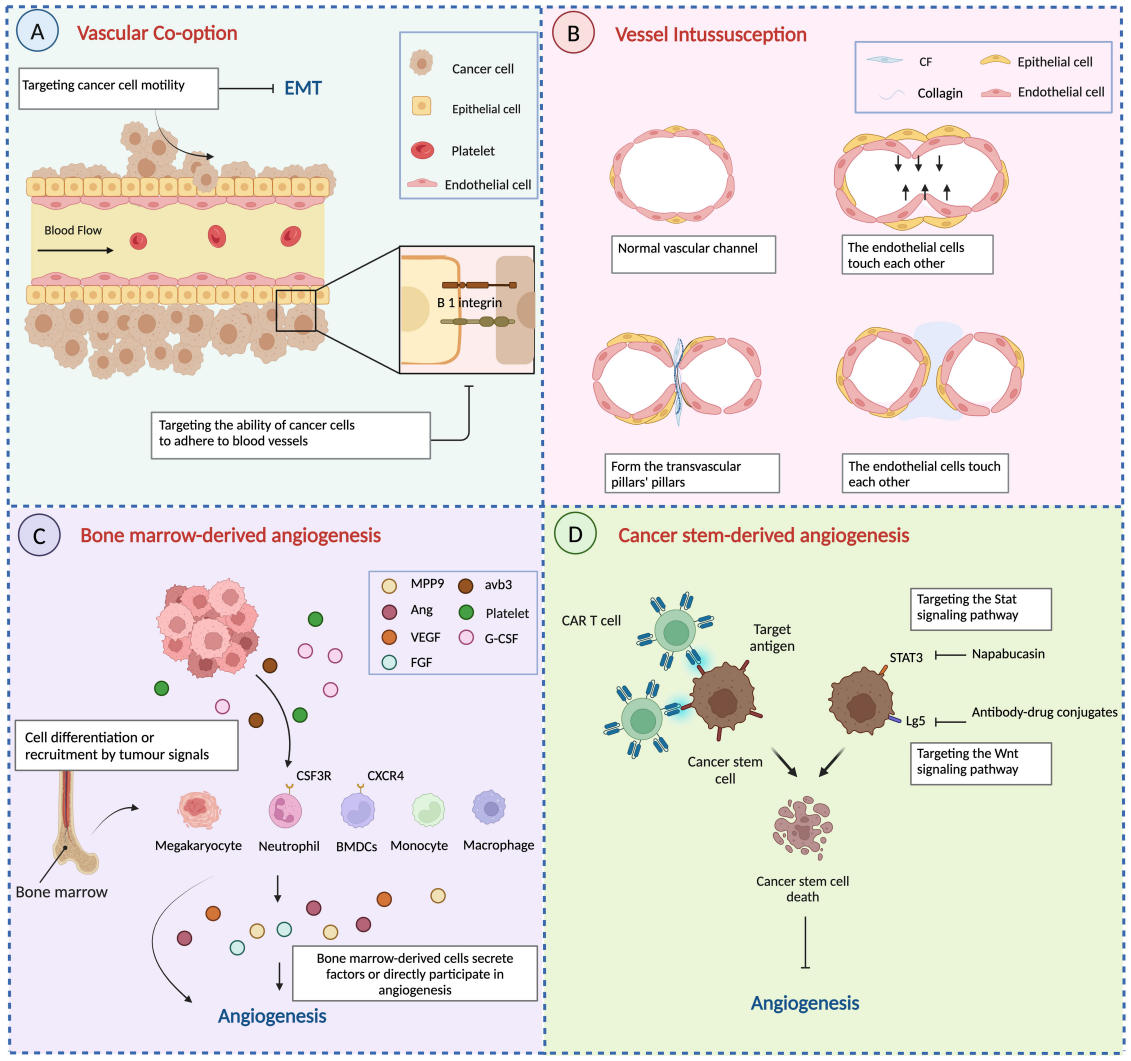
Other mechanisms of angiogenesis and anti-angiogenesis therapy. **(A)** Vascular co-option is prevented by reducing tumour cell EMT progression or targeting tumour cells’ propensity to adhere to blood vessels. **(B)** The process of vessel intussusception. **(C)** Bone marrow-derived cells develop into endothelial progenitor cells or are attracted around blood arteries to participate in angiogenesis directly or indirectly by secreting signalling chemicals. **(D)** Targeting aberrant signalling pathways in cancer stem cells and immunotherapy to reduce cancer stem cell angiogenesis. Created with BioRender.com.

Another widely studied change in vascular co-option is vascular adhesion. L1CAM, as a cell adhesion factor, is involved in the development of the nervous system and the progression of malignant tumours (51). The dynamic nature of L1CAM adhesion interactions may be particularly beneficial for cancer cells seeking cooperative vasculature when invading tissues, mediating and participating in vascular co-option of BMS cancer cells (35). L1CAM depletion significantly reduced the ability of H2030-BrM3 and MDA231-BrM2 cells to spread on the surface of the capillary cavity and decreased the activity of brain metastasis of cancer cells, thereby mediating co-option and metastatic growth of brain capillaries. Vascular co-option is prevented by reducing tumour cell EMT progression or targeting tumour cells’ propensity to adhere to blood vessels (**Figure 4A**).

### 2.3 Vessel-intussusception

In 1990, Burri discovered that capillary beds expanded by generating elongated columns of intravascular tissue in the lungs of rats and named this development pattern intussusceptive microvascular growth (IMG) (52). Since then, IMGs have been found in both embryonic and adult tissues and organs (53, 54). As research has progressed, IMGs have also been found to be involved in the abnormal proliferation of tumour vessels. Patan demonstrated that intussusceptive obstruction is an important mechanism of tumour angiogenesis by examining the growth of human colon adenocarcinoma (LS174T) (55). This is a mechanism that also occurs in several other tumours, such as colon (55, 56) and mammary carcinomas (57), melanoma (58), B-cell non-Hodgkin’s lymphoma (59), and glioma (60).

According to Ribatti, the steps in the development of intussusception are summarized as involving contact between neighbouring endothelial cell walls, reorganization of connections between ECs, central perforation between interconnected bilayers of ECs to form a core of mesenchymal pillars, and extension and invasion of the pillars by myofibroblasts and pericytes (8). Finally, the pillar increases in diameter, forming collagen fibres and, eventually, a capillary network (**Figure 4B**). Thus, in contrast to tumour sprouting angiogenesis, the most distinctive feature of intussusception is the absence of endothelial cell proliferation and the formation of ‘transvascular pillars’.

The mechanism underlying the onset and development of intussusceptive angiogenesis (IAs) is complex and dynamic, and no definitive studies exist that prove its development mechanism. Nevertheless, researchers have identified several factors that play key roles in this intricate process. First, shear stress generated by blood flow in the vasculature is an important aspect of IA development. Djonov demonstrated changes in branch morphology through blood pressure and blood clamping of a dichotomous branch in the developing chorioallantoic membrane (CAM) of the chick embryo (61). Second, VEGF-A is involved in SA and vascular co-option of tumour angiogenesis, and a considerable body of research indicates that the development of IA is also linked to VEGF (10). It is thought that shear stress triggers VEGF expression in endothelial cells adjacent to myofibers through the diffusion of nitric oxide in the vascular microenvironment, promoting tumour angiogenesis (62, 63). There is a correlation between the progression of intestinal obstruction and the production of VEGF in CAM micro-vessels. Inhibition of VEGF signalling pathway inhibits the development of capillaries that are dependent on intestinal obstruction (64). Furthermore, throughout the IA process, researchers have discovered that FGF2 increases the development of intraluminal pillars in ECs and pericytes (65). Observations have also shown that vascular morphological alterations through intussusceptive angiogenesis occur in hepatocellular carcinoma models during treatment with sirolimus, an inhibitor of the mammalian target of rapamycin (mTOR) pathway (7). The shift in angiogenesis from sprouting to intussusceptive might be an adaptive reaction to therapy using various antitumour and antiangiogenic drugs (8).

### 2.4 Vasculogenic mimicry

*In vitro*, researchers demonstrated the reproduction of the structured vascular channels present in human tumour tissue was shown in 1999, even in the absence of Ecs, in highly invasive melanoma cells (66). They named this mechanism vasculogenic mimicry. VM has been observed in numerous malignant tumours in recent years, including melanoma (67, 68), glioblastoma (69), HCC (70, 71), breast cancer (72, 73), and lung cancer (74). Unlike conventional angiogenesis, VM does not involve ECs and is characterized by tumour cells constituting vascular channels and the dense deposition of extracellular matrix (75). The presence of CD31/CD34-negative and PAS-positive cells and erythrocytes within the vasculature is considered a hallmark of VM (76). Saber provided microscopic and immunohistological data to facilitate the identification and understanding of *in vitro* and *in vivo* VM processes (77).

As previously mentioned in this review, hypoxia is a significant driver that not only affects tumour angiogenesis but also plays an essential role in antiangiogenic therapy (78, 79). HIF-1 binds to the HRE on the promoter sequences of Twist, Snail, and ZEB2 in the nucleus to regulate their expression. HIF-1 enhances LOXL2 expression in hepatocellular carcinoma, which promotes extracellular matrix remodelling and VM via the Snail/FBP1/VEGF pathway (80). A hypoxic microenvironment was observed to enhance VM in a melanoma mouse model compared to controls and was positively correlated with HIF-1α and HIF-2α (81). HIF-1 also controls the expression of other VM-related molecules, including VEGF, Twist, LOX, and matrix metalloproteinase (MMP). The expression of VEGF in melanoma has been reported to promote VM development by activating the PI3K/AKT pathway (82).

Knockdown of VEGF also reduces the expression of MMP and vascular endothelial-cadherin(VE-cadherin) (83). VE-cadherin erythropoietin-producing hepatocellular receptor A2 (Eph A2) plays an important role in VM. VE-cadherin regulates the phosphorylation level and localization of Eph A2, thereby activating focal adhesion kinase (FAK) and extracellular signal-regulated kinase (ERK) 1/2, and subsequently, activated Eph A2 can also activate the PI3K signalling pathway directly without relying on FAK and ERK1/2 (84). Furthermore, PI3K can upregulate MMP expression, which in turn activates MMP2, ultimately leading to the cleavage of laminin 5γ2 into the γ2’ and γ2x fragments, promoting extracellular matrix remodelling and VM formation (85, 86).

**Figure 5:**
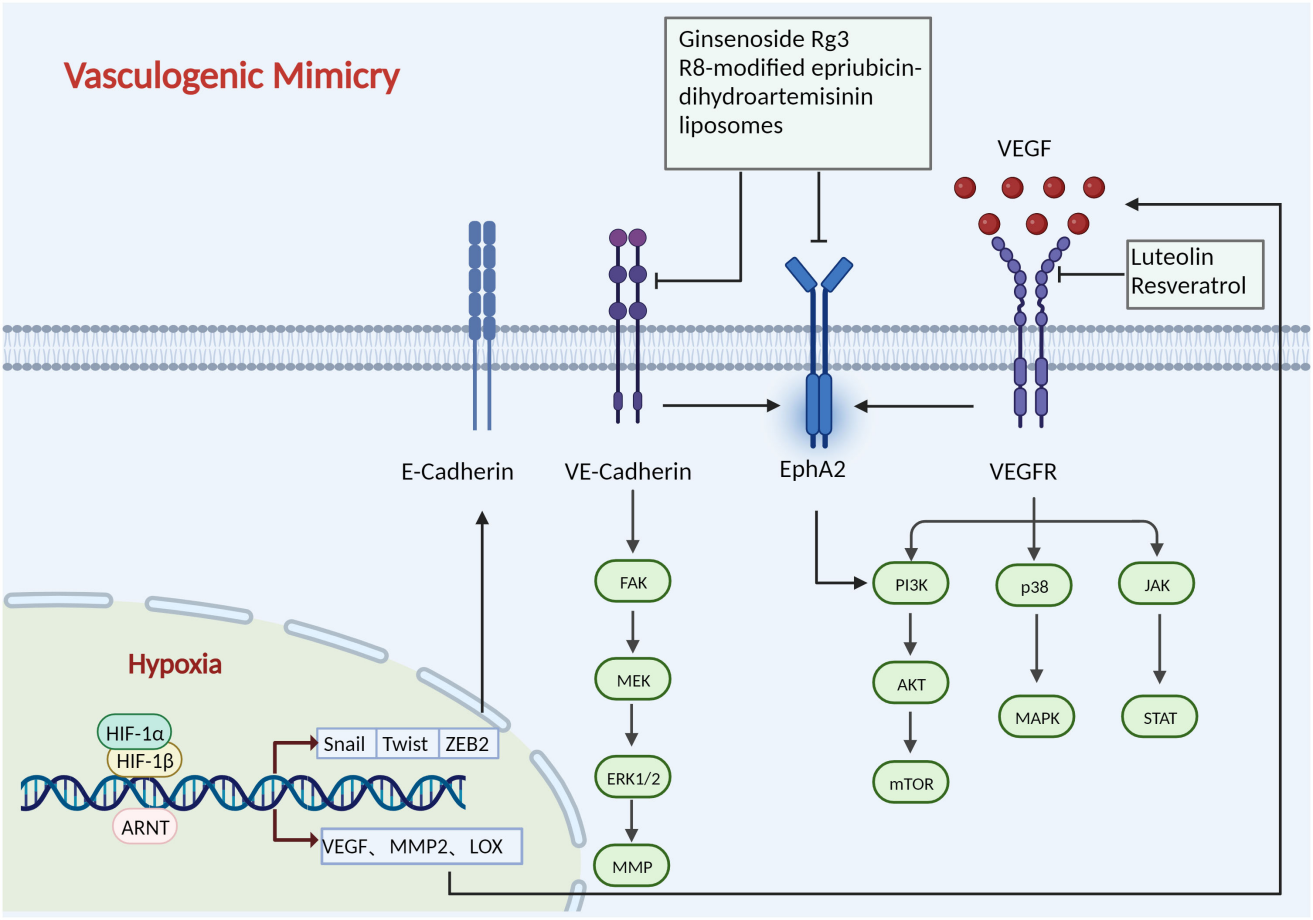
Mechanisms of vasculogenic mimicry. HIF-1 binds to the HRE on the promoter sequences of Twist, Snail, and ZEB2 in the nucleus to regulate their expression, which promotes extracellular matrix remodelling and VM. HIF-1 also controls the expression of other VM-related molecules, including VEGF, Twist, LOX, and MMP2. The expression of VEGF has been reported to promote VM development by activating the p38/MAPK, PI3K/AKT/mTOR, and JAK/STAT path-ways. VE-cadherin regulates the phosphorylation level and localization of Eph A2, thereby activating the PI3K/AKT, FAK/MEK/ERK1/2 pathways. Ginsenoside Rg3, R8-modified epirubicin dihydro-artemisinin liposomes inhibit VM progression by inhibiting the VE-Cadherin/EphA2/MMP9 signal axis. Luteolin and Resveratrol target VEGFR to inhibit angiogenesis. Created with BioRender.com.

Considerable progress has been made in inhibiting tumour angiogenesis by targeting the signalling pathways involved in VM (**Figure 5**). Ginsenoside Rg3 decreased the expression of VE-cadherin, EphA2, MMP9, and MMP2 to successfully block pancreatic cancer mimic angiogenesis (87). As a novel candidate for antitumour VM and anti-covariance treatment, miR-27b might bind to the 3’-untranslated region (3’UTR) of VE-cadherin mRNA to reduce the production of VE-cadherin in ovarian cancer cells (88). By suppressing STAT3 and PI3K/AKT, curcumin decreases the ability of hepatocellular carcinoma cells to simulate angiogenesis (89). By disrupting the PI3K/MMPs/Ln-52 signalling pathway, NCTD prevents tumour development and VM in human GBCs both *in vitro* and *in vivo* (90).

### 2.5 Bone marrow-derived angiogenesis

BMDCs play a crucial role in the pathogenesis and dissemination of malignancies due to their ability to suppress the immune system and promote the migration and vascularization of tumour cells. Comprising various cell types such as lymphocytes, stromal cells, bone marrow cells, and endothelial progenitor cells, BMDCs exhibit heterogeneity. They are recruited around blood vessels to actively participate in, either directly or indirectly through the secretion of signalling molecules (**Figure 4C**). Several haematopoietic populations from the bone marrow have been reported to contribute to tumour angiogenesis, such as monocytes (91), macrophages (92), granulocytes, and neutrophils (93, 94).

Daniel and his colleagues (95) used genetically labelled bone marrow progenitor cells and demonstrated, using high-resolution microscopy and flow cytometry that early-stage tumours recruit bone marrow-derived endothelial progenitor cells These endothelial progenitor cells differentiate into mature ECs, integrating into the developing tumour vasculature, thereby promoting tumour growth and progression. However, the mechanism by which tumours recruit BMDCs to the site of angiogenesis remains unknown. Tumours enhance the recruitment of BMDCs to the site of angiogenesis by secreting integrin avb3, a receptor that mediates cell−cell and cell–extracellular matrix adhesion (96). Platelets further mobilize and recruit CXCR4+ bone marrow-derived cells to promote vascularization (97). Tumours also induce myeloid cells to secrete factors associated with vascularization to promote tumour angiogenesis and progression. MMP-9, which controls the switch for angiogenesis by releasing VEGF from its membrane-bound form, has been shown to be expressed in myeloid cells (98). Rose demonstrated that HIF-1α partially induced the recruitment of bone marrow-derived CD45+ myeloid cells with Tie2+, VEGFR1+, CD11b+, and F4/80+ subpopulations, along with endothelial and pericyte progenitor cells, by increasing SDF1α and promoting the neovascularization of glioblastoma (99). Additionally, HIF-mediated negative regulation of PHD2 mobilizes BMDCs to regulate angiogenesis (100).

The recruitment of myeloid cells from bone marrow is directly associated with the emergence of tumour resistance or recurrence after antiangiogenic treatment. In response to antiangiogenic treatment, it has been demonstrated that macrophages increase the production of a number of angiogenic molecules, including FGF-1, FGF-2, MMP9, and Ang2 (101–104). Partially overcoming tumour resistance may be achieved by combining anti-VEGF with monotherapy targeting CD11b+Gr1+ myeloid cells (105). Thus, effective ablation of myeloid cells’ proangiogenic capacity may be achieved by extensively targeting them in cancer therapy (3). Inhibiting the gamma isoform of PI3K (PI3Kγ), which is largely expressed in myeloid cells and maintains their immunosuppressive and proangiogenic functions, maybe a promising targeting approach (106).

### 2.6 Cancer stem cell-derived angiogenesis

Cancer stem cells (CSCs) represent a small subset of cells within solid tumours capable of self-renewal and differentiation into various cell types present in the tumour. They play a crucial role in tumour metastasis, recurrence, and resistance to chemo and radiotherapy across a spectrum of cancers (107). Recent research has highlighted the role of CSCs in promoting angiogenesis through the release of angiogenic factors and exosomes (108–110). For instance, For example, CSCs in breast cancer have been found to secrete stromal cell-derived factor-1 and VEGF to facilitate angiogenesis (111). Further research discovered that enhanced IL-8 secretion by CD133+ CSCs in HCC not only induced tumour angiogenesis but also boosted tumour self-renewal and initiation (107). Additionally, emerging evidence suggests that tumour cells possess the ability to differentiate into phenotypes of ECs that are engaged in tumour angiogenesis. According to research on glioblastoma, ECs in varying quantities (range 20–90%, mean 60.7%) contain the same genetic abnormalities as tumour cells. This finding raises the possibility that GBM-derived stem cells can differentiate into ECs *in vitro* (112). This result suggests that tumour cells may have CSC origins, providing insight into the formation of VM.Studies by Sun et al. have shown that ALDH1+ and CD133+ breast cancer cells produced more VE-cadherin and generated VM channels (113). To support the formation of CSCs and preserve their stem properties, vascular niches in the tumour microenvironment also release growth factors through autocrine and paracrine pathways (107). Tumour growth is further encouraged by the positive feedback loop between tumour stem cells and angiogenesis.

From a therapeutic perspective, targeting CSCs is essential to impede tumour progression. The conventional approach involves blocking aberrant signalling pathways in CSCs, mainly including the Wnt, Nuclear factor-κB (NF-κB), Notch, Hedgehog, JAK/STAT, PI3K/AKT/mTOR, TGF/SMAD, and PPAR pathways. Napabucasin, a small-molecule inhibitor of STAT3, has shown efficacy in preventing the spread of biliary tract cancer, glioblastoma, and small-cell lung cancer (114–116). Clinical trials combining napabucasin with paclitaxel for gastric cancer have demonstrated favourable pharmacokinetic profiles, safety, and patient tolerability (117). Additionally, LGR5, a seven-transmembrane receptor from the G protein-coupled receptor family connected to the Wnt pathway that contains leucine-rich repeats, was first discovered as a marker of intestinal stem cells (118). LGR5 is a unique functional marker and a prognostic indicator that, by stimulating the Wnt pathway, can drive EMT and become a potential therapeutic target (119). In colon cancer, targeting LGR5 using antibody-drug conjugates greatly reduced tumour size and proliferation (120). Since normal human stem cells and tumour stem cells usually express the same surface and protein markers, therapeutic medication development faces significant challenges. **Table 1** summarizes some of the signature markers of tumour stem cells for reference. To boost antitumour efficacy, there is rising interest in combining immunotherapy with conventional chemotherapy and targeted medicines. The most promising immunotherapy is chimeric antigen receptor (CAR) T-cell treatment, which has been licensed for B-cell acute lymphoblastic leukaemia and B-cell lymphoma (144). CAR T cells targeting cd133 in glioma not only have good efficacy in xenograft models but also have no negative effect on normal human CD133+ haematopoietic stem cells (137). As a result, CART133 cells may be a useful treatment option for CSCs in different solid cancers.

**Table 1:** Summary of markers of different tumor stem cells.

Tumour Type	CSC Marker	Reference
Breast Cancer	CD44, CD55, CD133, ALDH1	(121–125)
Melanoma	CD133, ABCB5, CD20, CD271, SOX10	(122, 126–129)
Prostatic Cancer	CD44, CD133, a2β1	(122, 130)
Hepatocellular Carcinoma	CD133, EpCAM, CD90, CD13, SALL4	(122, 131–134)
Lung Cancer	CD133, CD44	(135, 136)
Glioblastoma	CD133, CD90, CD15, L1CAM	(137–140)
Colon Cancer	CD133, CD44, CD166, CD24	(141–143)

## 3 Clinical application of anti-angiogenic drugs

Anti-angiogenic therapy aims to inhibit tumour growth and metastasis by blocking the blood supply to tumour tissue using anti-angiogenic drugs. Clinically used anti-angiogenic drugs mainly include anti-angiogenic monoclonal antibodies (AA-MAs) and anti-angiogenic tyrosine kinase inhibitors (AA-TKIs). Compared with kinase inhibitors, monoclonal antibodies are artificially produced from hybridoma cells and have the advantages of high purity, high sensitivity, strong specificity, and low cross-reactivity. Tyrosine kinase inhibitors usually have multi-target effects and can interfere with cell signalling pathways and inhibit tumour growth (145). However, the utility of tyrosine kinase inhibitors is limited as not all cancers respond to them.

In addition to targeting the VEGF signalling pathway, there are also monoclonal antibodies targeting PDGF such as olaratumab. In 2016, the FDA approved olaratumab for the treatment of patients with soft tissue sarcoma. Combined treatment with doxorubicin and olaratumab significantly improves the overall survival of patients (146). Furthermore, some monoclonal antibodies targeting the Ang/TIE pathway are undergoing clinical trials. Faricimab, as a bispecific antibody, binds and inhibits both VEGF-A and Ang-2. Initially approved in 2022, it serves as a first-line treatment for neovascular age-related macular degeneration or diabetic macular edema (DME) (147). In addition to their use as monotherapies, monoclonal antibodies can serve as adjuvant therapy in surgery or in combination with chemotherapy, further expanding their potential in the anti-angiogenic treatment of tumours through ongoing clinical trials.

Sorafenib disrupts the downstream Ras/Raf/MEK/ERK pathway by inhibiting the autophosphorylation of Raf and kinase receptors, thereby impeding tumour angiogenesis and metastasis. In 2005 and 2007 respectively, it was approved by the FDA for the first-line treatment of RCC and advanced HCC (148, 149). In recent years, studies have found that it also shows anti-tumour activity in differentiated thyroid cancer (150). Sunitinib targets VEGFR-1/2/3, PDGFR, c-Kit receptor, fms-like tyrosine kinase-3 receptor (FLT-3), and receptor encoded by the ret proto-oncogene (Ret) and is approved for treatment drug-resistant gastrointestinal stromal tumours (GIST) and RCC (151–154). In 2009, the FDA approved pazopanib as a multikinase inhibitor against VEGFR-1/2/3, PDGFR-α/β, and c-Kit receptors for the treatment of patients with advanced RCC (155). In addition, pazopanib and olaratumab are both suitable for the treatment of advanced soft tissue sarcoma (156). Regorafenib, a small pan-tyrosine kinase inhibitor targeting VEGFR-1/2/3, PDGFR-α/β, FGFR-1/2, Tie2 and c-Kit receptors (157). Due to its excellent therapeutic effect in phase III clinical trials, it was also approved for the clinical treatment of metastatic RCC (158). Subsequently, regorafenib was approved for the treatment of HCC as a systemic approach that provided a survival benefit in patients with HCC that had progressed on sorafenib (159). Lenvatinib is a novel and potent tyrosine kinase inhibitor targeting VEGFR-1/2/3, PDGFR-α/β, FGFR-1/2/3, Ret and c-Kit (160). It has initially approved by the FDA for the treatment of radioactive iodine-refractory differentiated thyroid cancer depending on the trial, but more than 40% of patients who received lenvatinib had more adverse effects such as hypertension, diarrhoea, fatigue, decreased appetite, decreased weight, and nausea (161). Encouragingly, it still showed excellent anti-tumour activity in other tumours, renal cell carcinoma and metastatic hepatocellular carcinoma, so it was approved for treatment in 2015 and 2018 (162, 163). Of note, fewer patients treated with the combination of lenvatinib and everolimus experienced Grade 3 and 4 events compared with patients treated with lenvatinib alone (163). In addition to the aforementioned marketed and clinically evaluated antiangiogenic agents, several novel tyrosine kinase inhibitors such as nintedanib, anlotinib, and fruquintinib have shown strong antitumour activity in clinical trials, making them promising candidates for antiangiogenic therapy (164–167).

## 4 Anti-angiogenic therapy resistance and combination therapy

Mechanisms of resistance include the upregulation of alternative angiogenic pathways, recruitment of pro-angiogenic bone marrow-derived cells, and tumour cell adaptation to survive in a hypoxic environment. Overcoming resistance to anti-angiogenic therapy remains a significant challenge in cancer treatment. As mentioned above, tumours have different angiogenic mechanisms, and tumour cells provide a way to escape treatment, which increases the occurrence of drug resistance (168). In a recent study, it was discovered that VM formation is not contingent on the VEGF signalling pathway but rather on Foxc2, which promotes blood vessel formation by driving the ectopic expression of endothelial genes in tumour cells (169). Bevacizumab treatment also contribute cells to induce an endothelial phenotype through the IL8/CXCR2 pathway to promote VM initiation in GBM (170). Besides inherent drug resistance, vascular co-option may also lead to acquired resistance of metastases against angiogenic therapy (171). Vascular co-option mediates resistance to the anti-angiogenic drug Sunitinib in tumours with lung metastasis (34). Interestingly, vascular co-option was also associated with adverse effects of bevacizumab in patients with colorectal cancer liver metastases (168). Consequently, clinical treatment targeting the VEGF signalling pathway alone may not be sufficient, and selecting multi-target angiogenic tyrosine kinase inhibitors (TKIs) to counteract compensatory angiogenesis could represent a novel strategy (172).

Anti-angiogenic therapy targeting VEGF or VEGFR-2 has the potential to enhance T cell trafficking to tumours, thereby reducing immunosuppressive cytokines and regulatory T cells, which could help overcome resistance to checkpoint inhibitor therapy (173). The combination of antiangiogenic therapy and immunotherapy has demonstrated superior antitumour properties and significantly improved patient survival. In a phase III clinical trial of renal cell carcinoma, researchers found that the use of bevacizumab combined with the PD-1 inhibitor atezolizumab significantly improved the overall survival and progression-free survival rates of patients compared with sorafenib alone (174). Similarly, nivolumab (anti-PD-1 antibody) plus cabozantinib showed superior progression-free survival, overall survival, and objective response compared with sunitinib for the treatment of renal cell carcinoma (175). Although promising results have been reported with antivascular therapy combined with immunotherapy, some failures cannot be ignored. For instance, in patients with advanced non-squamous NSCLC, the combination of axitinib, paclitaxel, and carboplatin did not demonstrate improved efficacy compared to bevacizumab, paclitaxel, and carboplatin, and it was associated with poorer tolerability (94). Thus far, a large number of promising clinical studies are ongoing to explore the combination of immunotherapy and anti-angiogenic therapies as a new progression in tumour treatment (**Table 2**).

**Table 2:** Ongoing clinical trials of anti-angiogenic combination immunotherapy.

Trial Identifier	Treatment	Cancer Types	Phase	Date	Status
NCT02684006	Avelumab + Axitinib	mRCC	III	2016	Active, not recruiting
NCT03024437	Atezolizumab + Bevacizumab + Entinostat	RCC	II	2017	Suspended
NCT02366143	Atezolizumab+ Bevacizumab+ Chemotherapy	NSCLC	III	2018	Completed
NCT03463876	SHR-1210+ Apatinib	HCC、GC、EGJC	II	2019	Completed
NCT03434379	Atezolizumab+ Bevacizumab	HCC	III	2020	Completed
NCT03141177	Nivolumab+ Cabozantinib	RCC	III	2021	Active, not recruiting
NCT04732598	Atezolizumab + Bevacizumab + Paclitaxel	BC	III	2021	Active,not recruiting
NCT02811861	Pembrolizumab+ Lenvatinib	RCC	III	2021	Active, not recruiting
NCT05357417	Utidelone+ Bevacizumab	BC	II	2022	Recruiting
NCT05256472	Cadonilimab+ Axitinib	RCC	II	2022	Recruiting
NCT05485883	Tislelizumab+ Lenvatinib	RCC	II	2022	Recruiting
NCT06215651	Cadonilimab+ lenvatinib	HCC	II	2024	Not yet recruiting

Atezolizumab (anti-PD-L1 antibody); Pembrolizumab (anti-PD-1 antibody); Avelumab (anti-PD-L1 antibody); Nivolumab (anti-PD-1 antibody); SHR-1210 (anti-PD-1 antibody); Cadonilimab (anti-PD-1 antibody); Utidelone (anti-tubulin antibody).

## 5 Conclusions

Since Folkman proposed the idea of targeting angiogenesis as an anti-tumour therapy (1). Numerous studies have established a significant association between vascular mechanism switching and poor survival in clinical cancer patients, making it a potential predictor of poor prognosis (38, 164). Many anti-angiogenic therapies have been registered and developed to treat various tumours, mainly targeting the VEGF signalling pathway. However, it’s important to note that other non-sprouting angiogenic mechanisms, such as vascular co-option and vascular mimicry, are not entirely dependent on the VEGF signalling pathway. Moreover, in clinical practice, the occurrence of vascular mechanism conversion has been closely linked to resistance to antiangiogenic therapy (6). The existence of multiple angiogenesis mechanisms provides a new method for inhibiting tumour cell metastasis. While some small molecule drugs capable of targeting vascular co-option and vascular mimicry have been reported, **Table 3** lists some drugs and their applications that can effectively inhibit different types of angiogenesis mechanisms, but their efficacy lacks confirmation from clinical trials. Further exploration of different mechanisms can help scientists identify key molecular targets to develop more targeted drugs. Additionally, given the widespread application and clinical significance of anti-angiogenic therapy, there is a need to promote the development of new and more effective anti-angiogenic drug combination therapies to enhance efficacy. Understanding how different angiogenic mechanisms are switched during tumour development requires more research to determine whether different types of cancer and metastatic sites favour specific forms of angiogenesis, thereby enabling better management of these mechanisms. In summary, this review provides and highlights the need for a deeper understanding of the molecular mechanisms of tumour angiogenesis. This may facilitate the development of new and more effective anti-angiogenic drugs, bringing more clinical benefits to cancer patients and anti-tumour treatments.

**Table 3:** Summary of current drugs targeting angiogenesis.

Target Drug	Molecular Target or Function	Indications	References
Sprouting Angiogenesis			
Bevacizumab	VEGF-A	CRC, RCC, NSCLC, CC, GBM, OVC	(176)
Ramucirumab	VEGFR2	CRC, HCC, NSCLC, SA, GEJA	(177–179)
Sorafenib	VEGFR2, PDGFR, FGFR1	HCC, RCC, TC	(29, 180)
Regorafenib	VEGFR1/2/3, PDGFR	CRC, HCC, GIST	(179, 181, 182)
Sunitinib	VEGFR2, PDGF	RCC	(183)
Axitinib	VEGFR1/2/3, PDGFR	RCC	(184)
Apatinib	VEGFR2	SA, GEJA	(185)
Vascular co-option			
OS2966	β1 integrin	GBM	(36)
XAV939	Wnt/β-catenin	GBM	(186)
Vasculogenic mimicry			
Ginsenoside Rg3	VE−cadherin/EphA2/MMP9/MMP2	PAAD	(87)
Norcantharidin	PI3-K/MMPs/Ln-5γ2	GBC	(90)
Salinomycin	Rho-GTPases	BC	(187)
Luteolin	VEGF, Notch	GC	(12)
Resveratrol	VEGFR1/VEGFR2	MEL	(188)
Brucine	EphA2/MMP-2/MMP-9	BC	(189)
R8-modified epirubicin-dihydroartemisinin liposomes	VE-cadherin/TGF-β/MMP-2/HIF-1	NSCLC	(190)
Galunisertib	TGF-β1, Akt,	GBM	(191)
Dequalinium-modified paclitaxel plus ligustrazine micelles	VEGF, MMP2, TGF-β1, E-cadherin	NSCLC	(192)
SB225002	CXCR2	GBM	(170)
Entinostat	VEGF-A	BC	(193)
Verteporfin	Ang2, MMP2, VE-cadherin, α-SMA	PDAC	(194)
Niclosamide	miR-124, STAT3	OSCC	(195)

## Author contributions

RZ: Writing – review & editing, Writing – original draft. YY: Writing – review & editing. HG: Writing – review & editing. XH: Supervision, Writing – review & editing.
